# Elevated YKL40 is associated with advanced prostate cancer (PCa) and positively regulates invasion and migration of PCa cells

**DOI:** 10.1530/ERC-14-0267

**Published:** 2014-10

**Authors:** Varinder Jeet, Gregor Tevz, Melanie Lehman, Brett Hollier, Colleen Nelson

**Affiliations:** 1 Australian Prostate Cancer Research Centre – Queensland, Institute of Health and Biomedical Innovation, Queensland University of Technology, Princess Alexandra Hospital Translational Research Institute, Brisbane Australia; 2 Department of Urologic Sciences Vancouver Prostate Centre, University of British Columbia Vancouver, British Columbia Canada

**Keywords:** YKL40, prostate cancer, cell migration, cell invasion, metastasis, targeted therapy

## Abstract

Chitinase 3-like 1 (CHI3L1 or YKL40) is a secreted glycoprotein highly expressed in tumours from patients with advanced stage cancers, including prostate cancer (PCa). The exact function of YKL40 is poorly understood, but it has been shown to play an important role in promoting tumour angiogenesis and metastasis. The therapeutic value and biological function of YKL40 are unknown in PCa. The objective of this study was to examine the expression and function of YKL40 in PCa. Gene expression analysis demonstrated that *YKL40* was highly expressed in metastatic PCa cells when compared with less invasive and normal prostate epithelial cell lines. In addition, the expression was primarily limited to androgen receptor-positive cell lines. Evaluation of YKL40 tissue expression in PCa patients showed a progressive increase in patients with aggressive disease when compared with those with less aggressive cancers and normal controls. Treatment of LNCaP and C4-2B cells with androgens increased YKL40 expression, whereas treatment with an anti-androgen agent decreased the gene expression of *YKL40* in androgen-sensitive LNCaP cells. Furthermore, knockdown of *YKL40* significantly decreased invasion and migration of PCa cells, whereas overexpression rendered them more invasive and migratory, which was commensurate with an enhancement in the anchorage-independent growth of cells. To our knowledge, this study characterises the role of YKL40 for the first time in PCa. Together, these results suggest that YKL40 plays an important role in PCa progression and thus inhibition of YKL40 may be a potential therapeutic strategy for the treatment of PCa.

## Introduction

Each year, more than 258 000 men will die of prostate cancer (PCa), making PCa the second largest cause of cancer-related mortality in males globally (Jemal *et al*. 2011, Siegel *et al*. 2012). While early-stage PCa can be effectively treated by surgery or radiation therapy, metastatic PCa remains largely incurable. For locally advanced and metastatic disease, androgen deprivation therapy (ADT) is typically the first line of systemic therapy. Although ADT is effective initially in most patients, the cancer typically progresses within 12–36 months to castration-resistant prostate cancer (CRPC; Pagliarulo *et al*. 2012). Cytotoxics such as docetaxel and cabazitaxel, new androgen-targeted agents including abiraterone, enzalutamide, ARN-509 and orteronel, as well as new radiation agents such as RAD223 have provided significant clinical advances (Ezzell *et al*. 2013, Sridhar *et al*. 2014). However, despite these promising advances, CRPC remains a major clinical challenge and there is a compelling need for more effective therapies for patients with metastatic disease. The clinical development of new therapies is limited by the availability of informative biomarkers to identify patients who are likely to benefit from a particular type of therapy and by the question whether these markers can act simultaneously as a therapeutic target (Detchokul & Frauman 2011, Citrin *et al*. 2013). YKL40 is one such target that has shown promise in this area.

YKL40, also known as chitinase 3-like 1 (CHI3L1), is a secretory glycoprotein and a member of the ‘family 18 chitolectins’. YKL40 is produced by inflammatory cells and a variety of solid tumours, including breast, colon, lung, prostate, ovary and kidney tumours and glioblastoma (Culig *et al*. 2004). Several studies have correlated increased serum levels of YKL40 with the poor survival of cancer patients, suggesting its potential as a prognostic cancer biomarker (Jensen *et al*. 2003, Johansen *et al*. 2003, 2009, Bergmann *et al*. 2005). Evaluation of the serum levels of YKL40 and a clinical biomarker, C-reactive protein (CRP), demonstrated that elevated YKL40 levels are associated with an increased risk of gastrointestinal cancer, independent of CRP (Allin *et al*. 2012). In addition, patients with metastatic breast cancer had significantly higher serum concentration of YKL40 when compared with the control group (Jensen *et al*. 2003, Yamac *et al*. 2008). Further evidence suggests that YKL40 is more than a tumour biomarker and can function as a central player in the growth, invasion, metastasis and treatment resistance of cancer cells (Ku *et al*. 2011). Although the precise function of YKL40 is not clear, it is presumed to play a pivotal role in the proliferation and differentiation of cancer cells, support cell survival by activating protein kinase B (AKT) and inhibit apoptosis (Chen *et al*. 2011), stimulate angiogenesis (Faibish *et al*. 2011, Francescone *et al*. 2011), influence extracellular tissue remodelling (Johansen 2006) and act as a growth factor for fibroblasts (Recklies *et al*. 2002). YKL40 was also shown to promulgate the growth of breast and glioblastoma tumours by regulating angiogenesis, either independently or in coordination with the vascular endothelial growth factor axis (Shao *et al*. 2009, Francescone *et al*. 2011).

In PCa, higher serum levels of YKL40 have been reported in patients with primary PCa compared with those with benign prostate hyperplasia, suggesting that YKL40 may influence the progression and aggressiveness of PCa (Kucur *et al*. 2008). High serum YKL40 was also associated with shorter overall survival and early death in metastatic PCa patients undergoing hormonal therapy (Brasso *et al*. 2006, Johansen *et al*. 2007). More recently, investigations on the association of YKL40 with tumour burden and metastatic stage of PCa suggested that the elevated serum level of YKL40 may be a useful biomarker of increased tumour burden and invasiveness in patients with PCa and more informative than prostate-specific antigen (PSA) for predicting tumour burden and metastasis (Ozdemir *et al*. 2012). Despite these promising clinical studies, much more remains to be understood about the functional nature of YKL40. We therefore hypothesised that YKL40 may be a potential therapeutic target in PCa especially in metastatic CRPC and investigated its regulatory functions.

In this study, we focused on evaluating the biological role of YKL40 in PCa. We validated the expression of YKL40 in PCa cell lines and investigated whether it is regulated by androgens in PCa cells. We also determined whether the tissue expression of YKL40 correlates with the progression of clinical PCa. We further assessed the role of YKL40 in promoting the migration and invasion of PCa cells in addition to tumourigenicity.

## Materials and methods

### Cell culture

All cell lines were maintained in phenol red-free RPMI-1640 media supplemented with 5% foetal bovine serum (FBS; Life Technologies Australia (LTA)), except for LAPC4 cells, which were cultured in phenol red-free IMDM (LTA) supplemented with 10% FBS and 10^−8^ M dihydrotestosterone (DHT), and the human prostate epithelial cell line, RWPE-1, which was cultured in keratinocyte serum-free medium (LTA) supplemented with 50 μg/ml bovine pituitary extract and 5 ng/ml epidermal growth factor. LNCaP, 22RV1, PC3 and RWPE-1 cell lines were obtained from the American Type Culture Collection (ATCC, Rockville, MD, USA), whereas C42, C4-2B and LAPC4 cells were provided by Prof. Pamela Russell (Queensland University of Technology, Australia). Cells were maintained in a humidified incubator (5% CO_2_ and 95% O_2_) at 37 °C. Authenticity of cell lines was confirmed by short tandem repeat profiling (DDC Medical, Fairfield, OH, USA) and cells were routinely tested for mycoplasma.

### Hormone treatments

To model the androgen-deprivation conditions *in vitro*, cells were plated in FBS-containing medium and changed to 5% charcoal-stripped serum (CSS) media for 48 h, followed by treatment with optimised concentration of androgens DHT (10 nM final) or R1881 (1 nM final), and/or anti-androgen enzalutamide (10 μM final) for a further 48 h unless otherwise stated. Cells were incubated with enzalutamide 2 h before the addition of androgens.

### RNAi silencing and generation of stable cell lines

*YKL40* knockdown was achieved by RNAi, using siRNA specific to YKL40, as per the manufacturer's recommendations (Thermo Scientific Australia) for both LNCaP and C4-2B cell lines. To achieve the best knockdown, four different siRNAs were screened to select for the one showing the highest (≥70%) gene silencing efficiency 48 h after transfection with lipofectamine (LTA). YKL40 overexpression was achieved using the Precision LentiORF (pLOC) lentiviral vector system (Thermo Scientific Scoresby, Victoria, Australia). Lentiviral production was performed by co-transfection of pLOC vectors into 293T cells in the presence of pCMV-dR8.2 and pCMV-VSV-G packaging plasmids (Addgene, Cambridge, MA, USA). Cells were transduced with viral supernatants containing protamine sulphate (6 μg/ml) overnight and selected with the medium containing 10 μg/ml blasticidin.

### RT quantitative PCR

Total RNA was extracted using the RNeasy Mini System (Qiagen, Chadstone, Victoria, Australia) with on-column DNase treatment to remove contaminating DNA as per the manufacturer's guidelines (Qiagen). Samples were quantified by measuring the absorbance at 260 nm (Nanodrop, Wilmington, DE, USA). cDNA prepared from total RNA (first strand cDNA synthesis kit, LTA) was used to assess the expression of target genes using a SYBR Green Kit (LTA) in the 7900HT Fast Real Time PCR System (Applied Biosystems). The primers used were as follows: *YKL40* forward (f), 5′-cccaacctgaagactctcttg-3′ and *YKL40* reverse (r), 5′-ccaagatagcctccaacacc-3′; *AR* (f), 5′-ctggacacgacaacaaccag-3′ and *AR* (r), 5′-cagatcaggggcgaagtaga-3′ and *RPL32* (f), 5′-gcaacaaatcttactgtgccga-3′ and *RPL32* (r), 5′-gcattggggttggtgactct-3′. Samples were normalised to the housekeeping gene *RPL32* and then to the corresponding controls. Relative mRNA expression was analysed by the 2^−ΔΔ*C*t^ method (Livak & Schmittgen 2001).

### Immunoblotting

The protein expression of different genes was estimated by western blotting-based analysis. Cell culture medium was replaced with serum-free medium for 24 h and the supernatant was collected. Protein was concentrated from the cell culture supernatant using Amicon Ultra-2 centrifugal filter unit with ultracel-10 membrane (Merck Millipore, Kilsyth, Victoria, Australia). Whole cell lysates were prepared under non-denaturing conditions by treating cells with cell lysis buffer (Cell Signaling Technology (CST), Danvers, MA, USA) containing protease inhibitor cocktail (Roche, Castle Hill, New South Wales, Australia). All protein samples were quantified by the BCA assay kit (Pierce, Rockford, IL, USA). Briefly, an equal amount of protein was resolved by SDS/PAGE and transferred onto the nitrocellulose membrane. Blots were probed with primary antibodies, which are as follows: anti-CHI3L1 (1:500, R&D Systems, Minneapolis, MN, USA), anti-AR (1:1000, CST), β-tubulin (1:10 000, Sigma–Aldrich) and β-actin (1:10 000, CST). ECL-compatible secondary antibodies, anti-goat HRP (1:10 000, Santa Cruz Dallas, TX, USA), anti-mouse HRP and anti-rabbit HRP (1:10 000, GE Healthcare, Silverwater, New South Wales, Australia), were used. β-actin and β-tubulin were used as loading controls. Additionally, TGX stain-free gels (Bio-Rad, Gladesville, New South Wales), which show total protein content after SDS/PAGE, were used to validate equal protein loading. Proteins were visualised using an ECL Kit (Merck Millipore) on Bio-Rad's ChemiDoc XRS^+^ system with the image lab software. Densitometry analysis was performed on Bio-Rad's Image Lab Software (version 4.1).

### Evaluation of YKL40 expression in PCa patients

The human ‘Tissue Scan quantitative PCR (qPCR) array’ (HPRT502, HPRT503; Origene, Rockville, MD, USA) was used for determining the mRNA expression of *YKL40* in clinical samples of matched normal prostate and PCa tissues. The cancer specimens included various tumour–lymph node–metastasis (TNM) stages and Gleason grades of PCa. Tissue cDNAs of each array were synthesised by the manufacturer from high-quality total RNAs of pathologist-verified PCa tissues, normalised and validated with β-actin and provided with clinicopathological information, including age, sex, tumour stage, pTNM stage, etc. (Table 1 and Supplementary Table 1, see section on supplementary data given at the end of this article). RT-qPCR was performed as explained above. Gene expression was normalised to the housekeeping gene β-actin (forward- and reverse-) supplied with the array kit.

### Cell migration assays

Monolayer wound-healing cell migration assays were performed using a real-time cell imaging system (Incucyte, Essen Bioscience (EB), Ann Arbor, MI, USA) in which cells are imaged inside a standard incubator under optimal physiological conditions for the entire duration of the experiment. Briefly, 96-well image lock plates (EB) were coated overnight with poly-ornithine (40 μl, Sigma) for improving cell adherence and LNCaP and C4-2B cells (2.5×10^4^) were plated the following day. Cells were grown to confluence (usually 24 h) and treated with an optimised dose of anti-proliferation agent, mitomycin C (20 μM, Sigma), for 2 h before scratching to neutralise the effect of proliferation on cell migration. Scratches were made with a 96-pin wound maker (EB) and the wells were washed with PBS to remove any debris before replenishment with 100 μl media containing 5% CSS. For *YKL40* siRNA experiments, 1.5×10^4^ cells were plated overnight to achieve 50–60% confluency followed by incubation with an optimised concentration of either siRNA or scrambled non-specific siRNA controls in 100 μl serum-free media for 4 h, and then 5 μl CSS was added on top (i.e. 5% CSS/well). Cells were allowed to form a confluent monolayer after transfection (up to 72 h) and then scratched and imaged. The wound closure was quantified by an integrated metric: relative wound density (RWD). This metric relies on measuring the spatial cell density in the wound area relative to the spatial cell density outside of the wound area at every time point, thus RWD is self-normalising for the changes in wound density, which may occur outside the wound due to cell proliferation and/or pharmacological effects (EB). Images captured at 2 h intervals were collated into video files using the Incucyte imaging software. All samples were evaluated in six replicates in four independent experiments.

### Invasion assays

LNCaP and C4-2B cells (2.5×10^4^) were grown on the image lock plates coated overnight with Matrigel (BD Biosciences, San Jose, CA, USA; diluted to 100 μg/ml in serum-free media) and a wound was induced with the wound maker as explained above, following which another layer of Matrigel (1 mg/ml) was added on top. Cells were prepared as explained in the preceding section. Time-lapse images of cells penetrating through Matrigel were acquired and data were monitored and quantified using the Incucyte Software as described above. All samples were evaluated in six replicates in three independent experiments.

### Anchorage-independent growth assays

Anchorage-independent growth was evaluated by the soft agar colony count assay in three different experiments. Cells were seeded (5×10^3^ cells/well) in triplicate in six-well plates. Colonies (≥20 cells) were counted after 14 days using a dissecting microscope. Briefly, a base layer of agar was prepared by mixing equal volumes of 1.2% agarose at 37 °C, and pre-warmed 2× DMEM with 10% FBS poured onto a six-well plate (2 ml/well) and allowed to polymerise overnight in the incubator. The top agar layer was made by mixing equal volumes of warm 0.6% agarose and 2× DMEM with 10% FBS-containing 5000 cells from each experimental cell line (2 ml/well). Top agar containing cells was poured onto the base agar and the plates were incubated at 37 °C/5% CO_2_ and media (0.5 ml) were replenished every 3 days. Colonies were counted by manually drawing squares underneath the plate for visible demarcation and the colony count was plotted from the average of 16 different fields per well.

### Statistical analysis

Two-tailed Student's *t*-test with the Mann–Whitney *U* test and one-way ANOVA with Tukey's multiple comparison tests were used to analyse the results (GraphPad Prism 5, La Jolla, CA, USA). The *P* value of ≤0.05 was considered significant. Results are representative of at least three independent experiments with triplicate samples, unless stated otherwise.

## Results

### Expression of *YKL40* in PCa cell lines

**Figure 1:**
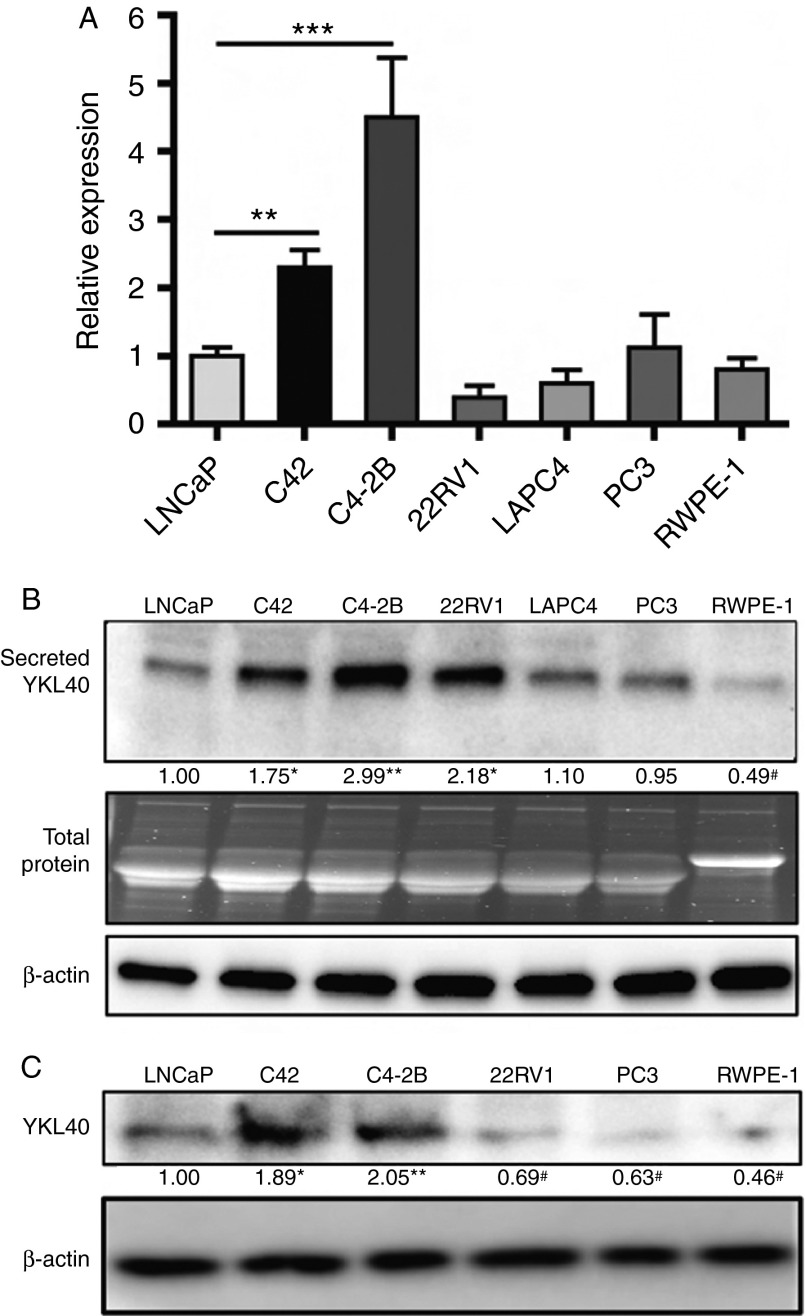
Expression of YKL40 in PCa cell lines: (A) RT-qPCR analysis showing the gene expression of *YKL40* in PCa cell lines. All samples were normalised to the corresponding housekeeping gene, *RPL32*, and then normalised relative to LNCaP. All samples were evaluated in triplicate and the average values of the three independent experiments are expressed as mean±s.e.m. (B) Secreted levels of YKL40 protein (40 kDa) were evaluated by culturing cells in the serum-free medium for 24 h, following which protein was concentrated from the supernatant and equal amounts were resolved by immunoblotting. TGX stain-free gels were used to represent total protein loading from cell culture supernatants. Additionally, β-actin (45 kDa) was used as an internal control. Data represent the average of three independent experiments. (C) Intracellular expression of YKL40 was determined by western blotting in PCa cell lines with β-actin serving as the internal control. Data represent the average of three independent experiments. Densitometry values for YKL40, normalised to β-actin and presented relative to LNCaP (first lane; set as onefold), are included below the lanes. *P<0.05, **P<0.01, ***P<0.001 and ^#^ *P*<0.05.

We first evaluated the gene expression of *YKL40* in a panel of PCa cell lines. We found that C42 and C4-2B cells, more invasive derivatives of LNCaP cell line, expressed significantly higher levels of *YKL40* mRNA, when compared with LNCaP cells. Other androgen receptor (AR)-positive cell lines, 22RV1 and LAPC4, AR-negative (AR^−ve^) PC3 cells and the normal prostate epithelial cell line, RWPE-1, did not show any significant difference in the expression levels of *YKL40* mRNA, in comparison to LNCaP cells (Fig. 1A). In agreement with the gene expression findings, the secreted protein levels of YKL40 in cell culture supernatants were highest in C4-2B cells, followed by C42 and LNCaP, and lowest in LAPC4, PC3 and RWPE-1 cells (Fig. 1B). Furthermore, we assessed the intracellular protein expression of YKL40 in whole cell lysates of cell lines and observed that the results correlated with the mRNA expression levels (Fig. 1C). Although 22RV1 cells displayed relatively high levels of secreted YKL40 (Fig. 1B), the intracellular protein expression correlated with mRNA expression (Fig. 1C). These results taken together suggest that YKL40 expression increased with progression to CRPC phenotype in AR^+ve^, hormonally responsive cell lines.

On the basis of these results, we decided to use LNCaP and C4-2B cells for subsequent studies, as they represent the progression of PCa disease from androgen-dependent disease (LNCaP) to CRPC bone metastatic disease (C4-2B), while remaining hormonally responsive (Thalmann *et al*. 1994, 2000).

### Androgen treatment induces the expression of YKL40

**Figure 2:**
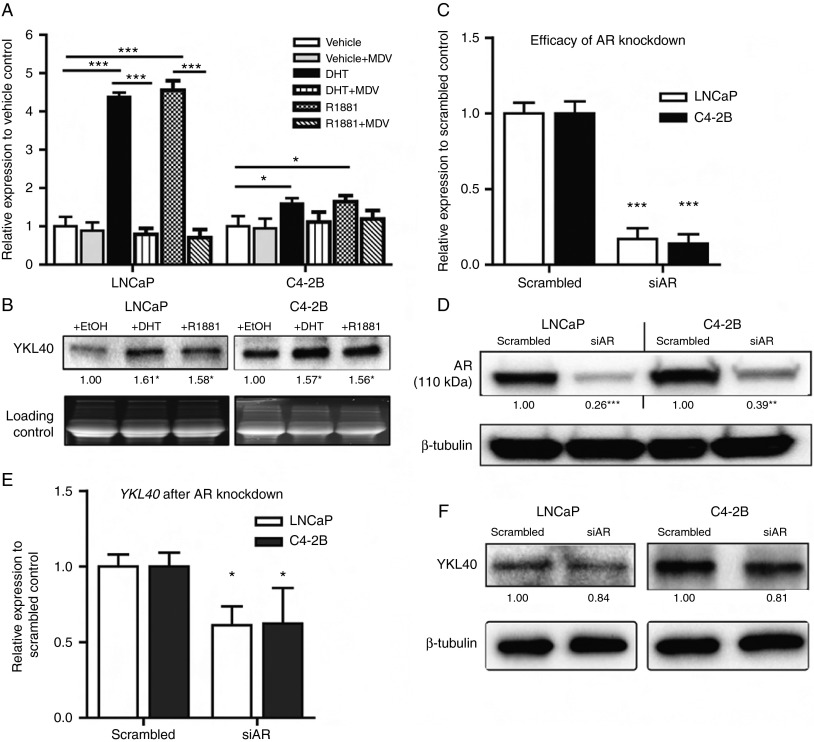
Androgen receptor-mediated regulation of YKL40 expression in LNCaP and C4-2B cell lines: (A) RT-qPCR analysis showing the effect of androgens (DHT and R1881) and anti-androgen enzalutamide (Enz) on *YKL40* gene expression. All samples were normalised to the corresponding housekeeping gene, *RPL32*, and then normalised relative to the vehicle (ethanol) control (mean±s.e.m., *n*=3 for three separate experiments). (B) Secreted levels of YKL40 after treatment with androgens were determined by western blotting. TGX stain-free gel was used as a loading control. Data represent the average of three independent experiments. RT-qPCR and immunoblotting showing the efficacy of AR knockdown (siAR) at the gene (C, mean±s.e.m., *n*=3) and protein (D, *n*=3) levels. (E) Effect of *AR* silencing (siAR) on *YKL40* gene expression as evaluated by RT-qPCR (mean±s.e.m., *n*=3). (F) Representative western blot showing the effect of AR protein knockdown on YKL40 protein expression. β-tubulin (50 kDa) served as an internal control (*n*=3). Densitometry values for specific proteins, normalised to loading controls and presented relative to vehicle controls (set as onefold), are included below the lanes. *P<0.05, **P<0.01 and ***P<0.001.

As the expression of YKL40 correlated with the progression to castration resistance in AR^+ve^ PCa cells, we then determined whether YKL40 was regulated by androgens. This was achieved by evaluating the gene expression of *YKL40* in the presence and absence of androgens (DHT and R1881) and the anti-androgen agent (enzalutamide) using RT-qPCR. We found that *YKL40* mRNA was significantly elevated in LNCaP cells in response to both 10 nM DHT (4.30-fold, *P*<0.001) and 1 nM R1881 (4.55-fold, *P*<0.001). This response was blunted in C4-2B cells, which showed small but significant responses to both DHT (1.59-fold, *P*<0.05) and R1881 (1.65-fold, *P*<0.05) (Fig. 2A). Furthermore, the androgen-induced increase in *YKL40* mRNA was significantly abrogated upon treatment with 10 μM enzalutamide in LNCaP cells, whereas C4-2B cells did not show any significant reduction (Fig. 2A). The weak androgen response in C4-2B cells is probably due to their high baseline expression of androgen-activated genes in these cells. The upregulation of *YKL40* mRNA with androgen treatment was accompanied by a commensurate increase in secreted YKL40 protein levels (Fig. 2B). When compared with the vehicle control, YKL40 protein was significantly upregulated in both DHT- and R1881-treated LNCaP cells (*P*<0.05). Similarly, C4-2B cells displayed a significant upregulation of YKL40 protein levels following treatment with either DHT or R1881 (*P*<0.05; Fig. 2B).

To further examine whether androgen induction of YKL40 is mediated by AR, AR-targeted siRNA was used to suppress AR gene and protein expression (Fig. 2C and D). We found that the mRNA expression of *YKL40* was significantly downregulated after *AR* knockdown in both LNCaP and C4-2B cell lines (Fig. 2E, *P*<0.05). However, while we observed a reduction in the YKL40 protein expression after *AR* knockdown, this decrease was not significant (Fig. 2F). This finding indicates that although YKL40 is responsive to androgens, there may be other mechanisms contributing to YKL40 production. This seems to be especially true for C4-2B cells, which, despite their positive AR status, also show castration resistance.

### Expression of YKL40 in PCa patients

**Figure 3:**
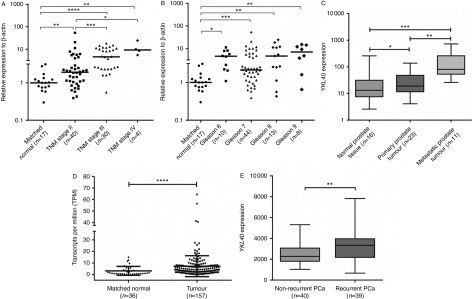
*YKL40* gene expression in matched normal and PCa tissues: (A) gene expression of *YKL40* was determined in PCa patients representing progressive TNM stages when compared with the matched normal prostate tissue using RT-qPCR. Data are expressed as median values of three independent experiments. (B) RT-qPCR analysis showing *YKL40* mRNA expression in different Gleason grades of PCa when compared with the control tissue. Data represent the median values of three independent experiments. (C) Expression of YKL40 in primary and metastatic PCa cells when compared with the corresponding normal samples in microarray data from publicly available dataset GDS2545 (Chandran *et al*. 2007). Data are expressed as median values. (D) Analysis of RNA-sequencing data derived from ‘The Cancer Genome Atlas’ dataset showing the expression of YKL40 in normal vs tumour specimens. Data are expressed as median values. (E) Gene expression of *YKL40* was validated in GDS4109 (Sun & Goodison 2009) samples that comprise microarray analysis of recurrent vs non-recurrent PCa. Data represent the median values. *P<0.05, **P<0.01, ***P<0.001 and ****P<0.0001.

After elucidating the YKL40 expression patterns in the *in vitro* model system, we examined the expression levels in PCa patient samples. Although elevated serum protein levels of YKL40 have been reported in PCa patients, no data are available regarding the tissue specific expression of YKL40 in PCa. To investigate this, we utilised commercial cDNA arrays encompassing various TNM stages and Gleason grades of the disease (Table 1 and Supplementary Table 1). When compared with the matched normal controls, significantly higher expression of YKL40 was found in TNM stage II (*P*<0.01), stage III (*P*<0.0001) and stage IV patients (*P*<0.01) (Fig. 3A). Differences between the stages were also observed as TNM stage III (*P*<0.001) and stage IV (*P*<0.05) patients had significantly higher levels of YKL40 compared with TNM stage II patients (Fig. 3A). We then categorised these patients on the basis of Gleason grades and found that Gleason grade 7, 8 and 9 tumour tissues had significantly higher levels of YKL40 when compared with the normal controls (Fig. 3B). However, we did not find any significant differences among the Gleason grades (Fig. 3B). *In silico* analysis of *YKL40* mRNA expression using publicly available gene expression data (Gene Expression Omnibus datasets (GDS), http://www.ncbi.nlm.nih.gov/gds) found *YKL40* gene expression to be significantly increased in patients with metastatic PCa when compared with primary PCa and normal prostate tissue (Fig. 3C, GDS2545; Chandran *et al*. 2007). Furthermore, a significant difference between primary PCa and normal tissue was also measured (Fig. 3C, GDS2545; Chandran *et al*. 2007). We also extracted information from the publicly accessible ‘The Cancer Genome Atlas (TCGA)’ RNA-sequencing dataset and evaluated the expression of YKL40 in normal adjacent prostate tissue vs prostate tumour tissues. TCGA results showed that *YKL40* mRNA is significantly increased in PCa tumour tissue when compared with the non-cancerous tissue (Fig. 3D). Additional analysis revealed higher expression of YKL40 in patients with biochemically recurrent (three consecutive increases in the serum level of PSA after radical prostatectomy) PCa disease when compared with those with non-recurrent disease (Fig. 3E, GDS4109; Sun & Goodison 2009), which also corroborates the trends observed at the protein level in a previous study (Johansen *et al*. 2007). Our findings, coupled with previous reports, firmly highlight the potential role of YKL40 in the PCa disease progression.

### Characterising the functional role of YKL40 in PCa cells: determination of migration, invasion and anchorage independence

**Figure 4:**
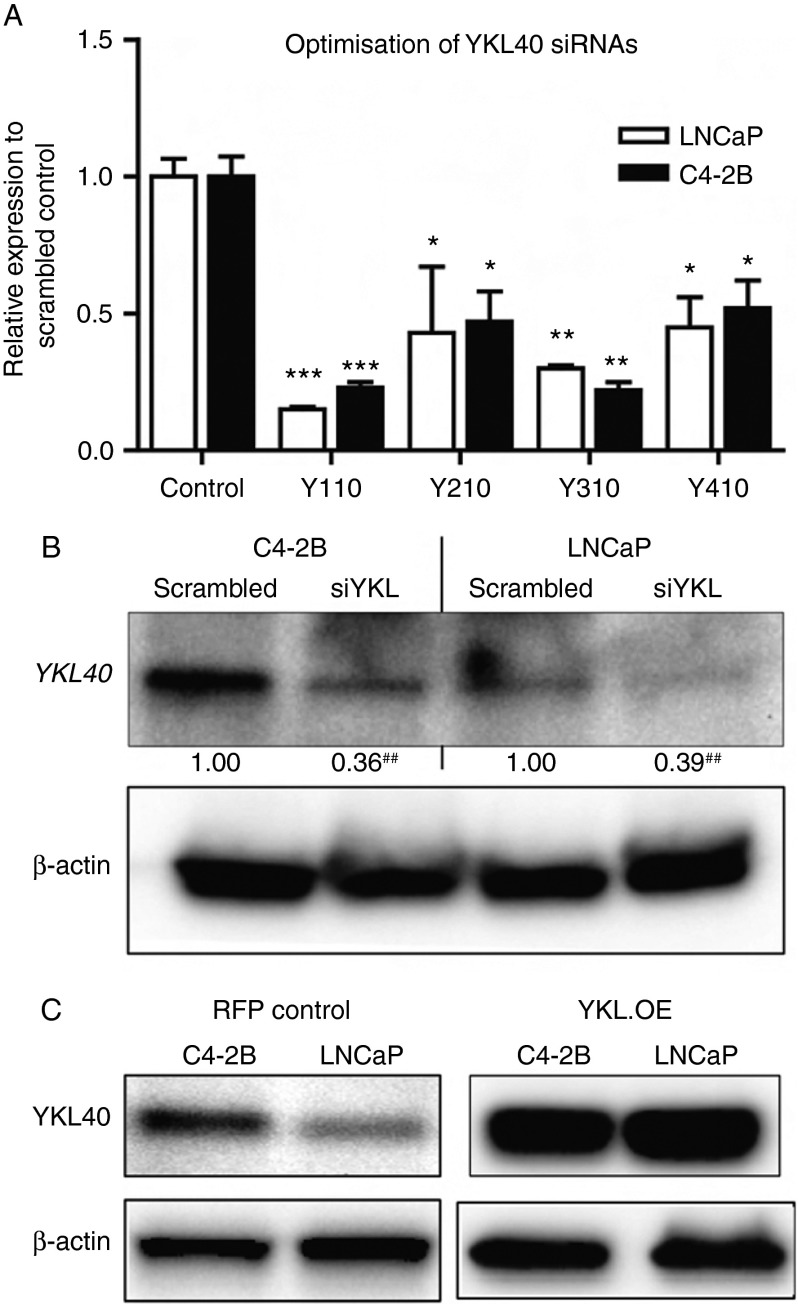
Knockdown and overexpression of *YKL40* in PCa cells: (A) the optimisation of *YKL40* knockdown to select for the siRNA showing the maximum knockdown as determined by RT-qPCR (mean±s.e.m., *n*=3). *P<0.05, **P<0.01 and ***P<0.001. (B) The confirmation of *YKL40* knockdown at the translational level by western blotting (*n*=3). ^##^ *P*<0.01 (vs scrambled control). (C) The representative western blot of stable overexpression of YKL40 in control (RFP) and YKL40-overexpressing (YKL.OE) cells.

Previous studies have supported a functional role for YKL40 in promoting metastasis, invasion and angiogenesis in multiple tumour types. Results from our analysis of the LNCaP–C4-2B progression model and the *in silico* gene expression of clinical specimens indicate that YKL40 is consistently upregulated in advanced PCa. We therefore sought to investigate whether YKL40 plays a functional role in biological phenotypes relevant to the invasion and metastasis of PCa cells. To achieve this, we first tested four individual siRNA sequences specific to YKL40 (Y110, Y210, Y310 and Y410) to identify the optimal siRNA for suppression of endogenous YKL40 expression in LNCaP and C4-2B cell lines (Fig. 4A). The siRNA clone Y110 (subsequently referred to as siYKL) was found to efficiently suppress YKL40 mRNA (Fig. 4A) and protein expression (Fig. 4B) in both cell lines. LNCaP and C4-2B cell lines overexpressing YKL40 (YKL.OE) were generated via the stable expression of the YKL40 open reading frame using the pLOC lentiviral vector. Cell lines stably expressing red fluorescent protein (RFP) were also generated as controls. The overexpression of YKL40 in LNCaP and C4-2B cells was confirmed by western blotting (Fig. 4C).

### YKL40 increases the migratory potential of PCa cells

**Figure 5:**
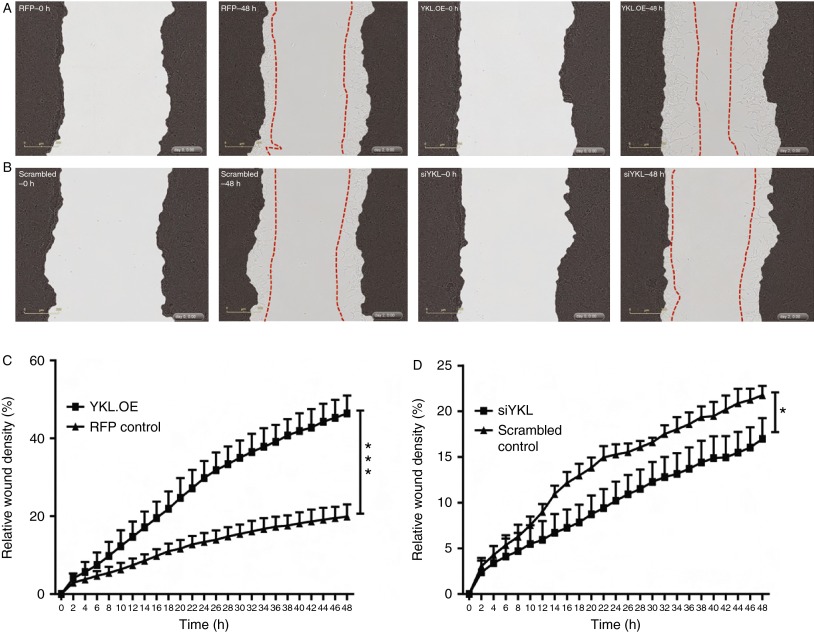
YKL40-mediated cell migration in LNCaP cells: (A) stably transduced LNCaP cells were grown to confluence and a wound was made using a 96-well wound maker and images were recorded over 2 days. Representative images are shown for RFP control cells at time points – 0 h (RFP–0 h) and 48 h (RFP–48 h). Similarly, YKL40-overexpressing (YKL.OE) LNCaP cells were scratched and real-time images were captured over 48 h. Representative images are shown at 0 h (YKL.OE–0 h) and 48 h (YKL.OE–48 h). (B) Scrambled control and *YKL40*-silenced cells (siYKL) were scratched and representative images are shown at time points – 0 h (scrambled–0 h, siYKL–0 h) and 48 h (scrambled–48 h, siYKL–48 h). Red dotted lines outline the extent of migration. The percentage of cells that migrated through the wounded area was plotted as relative wound density in RFP control vs YKL.OE cells (C) and scrambled control vs siYKL cells (D). Data represent mean±s.e.m., *n*=4. Photomicrographs were taken at 10× magnification. *P<0.05 and ***P<0.001. A full colour version of this figure is available at http://dx.doi.org/10.1530/ERC-14-0267.

**Figure 6:**
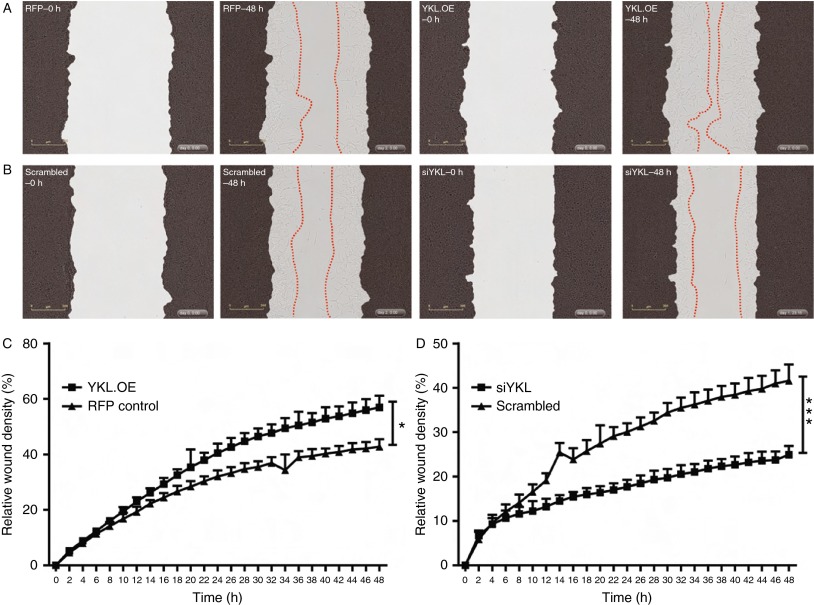
YKL40-mediated cell migration in C4-2B cells: (A) stably transduced cells were grown to confluence and a wound was made by the 96-well wound maker and images recorded over two days using time-lapse microscopy. Representative images are shown for RFP control cells at time points – 0 h (RFP–0 h) and 48 h (RFP–48 h). Similarly, YKL40-overexpressing (YKL.OE) C4-2B cells were scratched and real-time images were captured over 48 h. Representative images are shown at 0 h (YKL.OE–0 h) and 48 h (YKL.OE–48 h). (B) Scrambled control and *YKL40*-silenced cells (siYKL) were scratched and representative images are shown at time points – 0 h (scrambled–0 h, siYKL–0 h) and 48 h (scrambled–48 h, siYKL–48 h). Red dotted lines outline the extent of migration. The percentage of cells that migrated through the wounded area was plotted as relative wound density in RFP control vs YKL.OE cells (C) and scrambled control vs siYKL cells (D). Data represent mean±s.e.m., *n*=4. Photomicrographs were taken at 10× magnification. *P<0.05 and ***P<0.001. A full colour version of this figure is available at http://dx.doi.org/10.1530/ERC-14-0267.

YKL40 has previously been reported to induce migration of endothelial (Malinda *et al*. 1999) and glioblastoma cells (Ku *et al*. 2011) *in vitro*. As a higher rate of cell migration is a hallmark of aggressive cancers, we postulated that YKL40 might induce the migration of PCa cells. To investigate this possibility, we monitored the effect of YKL40 on the migratory potential of PCa cells using monolayer wound-healing assays. We found that overexpression of YKL40 in LNCaP cells (YKL.OE) significantly enhanced their ability to migrate into the wounded area (*P*<0.001) (Fig. 5A and C; Supplementary Movies 1 and 2, see section on supplementary data given at the end of this article). By contrast, the suppression of endogenous YKL40 expression using siYKL led to a small but significant reduction in the LNCaP wound closure (*P*<0.05; Fig. 5B and D; Supplementary Movies 3 and 4). In C4-2B cells, the effect of *YKL40* knockdown on the rate of migration was much more pronounced compared with that observed in LNCaP cells and resulted in a significant reduction in the percentage of migrating cells (*P*<0.001; Fig. 6A and C; Supplementary Movies 5 and 6). Confirming the results observed in LNCaP cells, the overexpression of YKL40 in C4-2B cells (YKL.OE) led to a significant increase in C4-2B cell migration (*P*<0.05) (Fig. 6B and D; Supplementary Movies 7 and 8).

### YKL40 increases the invasive potential of PCa cells

**Figure 7:**
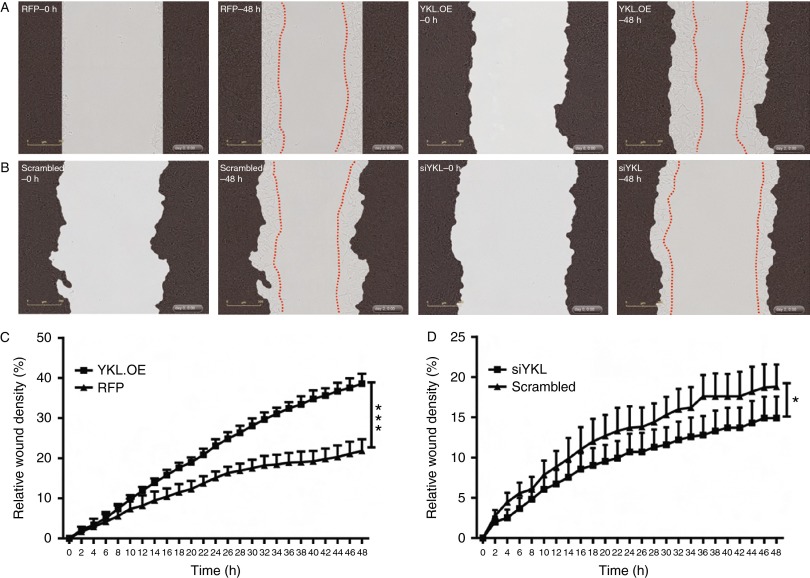
YKL40-mediated cell invasion in LNCaP cells: (A) cells were layered onto Matrigel-coated plates and the wound was made using the wound maker followed by adding another layer of Matrigel on top. Representative images are shown at 0 h (RFP, YKL.OE–0 h) and 48 h (RFP, YKL.OE–48 h). (B) Scrambled control and *YKL40*-silenced cells (siYKL) were scratched and representative images are shown at time points – 0 h (scrambled–0 h, siYKL–0 h) and 48 h (scrambled–48 h, siYKL–48 h). Red dotted lines outline the extent of invasion. The percentage of cells that invaded through the wounded area was plotted as relative wound density in RFP control vs YKL.OE cells (C) and scrambled control vs siYKL cells (D). Data are expressed as mean±s.e.m., *n*=3. Photomicrographs were taken at 10× magnification. *P<0.05 and ***P<0.001. A full colour version of this figure is available at http://dx.doi.org/10.1530/ERC-14-0267.

**Figure 8:**
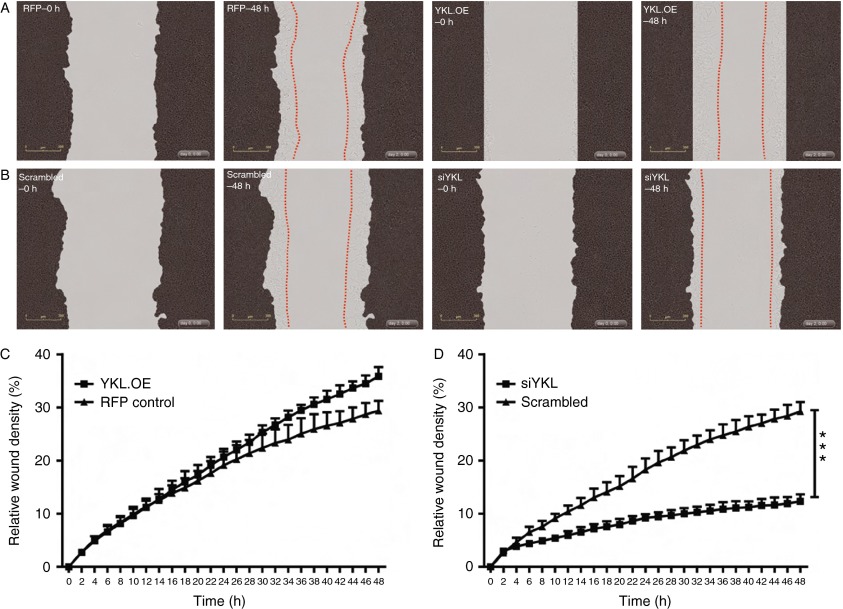
YKL40-mediated cell invasion in C4-2B cells: (A) C4-2B cells were layered onto Matrigel-coated plates and the wound was made using the wound maker followed by adding another layer of Matrigel on top. Representative images are shown at 0 h (RFP, YKL.OE–0 h) and 48 h (RFP, YKL.OE–48 h). (B) Scrambled control and *YKL40*-silenced cells (siYKL) were scratched and representative images are shown at time points – 0 h (scrambled–0 h, siYKL–0 h) and 48 h (scrambled–48 h, siYKL–48 h). Red dotted lines outline the extent of invasion. The percentage of cells that invaded through the wounded area was plotted as relative wound density in RFP control vs YKL.OE cells (C) and scrambled control vs siYKL cells (D). Data are expressed as mean±s.e.m., *n*=3. Photomicrographs were taken at 10× magnification. ***P<0.001. A full colour version of this figure is available at http://dx.doi.org/10.1530/ERC-14-0267.

Given that YKL40 is known to drive tumour cell invasiveness (Ku *et al*. 2011, Singh *et al*. 2011), we performed invasion assays to determine the role of YKL40 in the invasion of cells through Matrigel. In concert with the cell migration data, we found that YKL40 overexpressing LNCaP cells displayed significantly increased invasion through Matrigel when compared with the RFP control cells (*P*<0.001; Fig. 7A and C; Supplementary Movies 9 and 10, see section on supplementary data given at the end of this article). By contrast, the suppression of YKL40 expression in LNCaP cells (siYKL) led to reduction in the number of invading cells (*P*<0.05) (Fig. 7B and D; Supplementary Movies 11 and 12). Similarly, the suppression of YKL40 expression in C4-2B cells significantly reduced cell invasion (*P*<0.001; Fig. 8A and C; Supplementary Movies 13 and 14), while overexpression of YKL40 did not yield a significant change (Fig. 8B and D; Supplementary Movies 15 and 16).

### YKL40 promotes clonal growth *in vitro*

**Figure 9:**
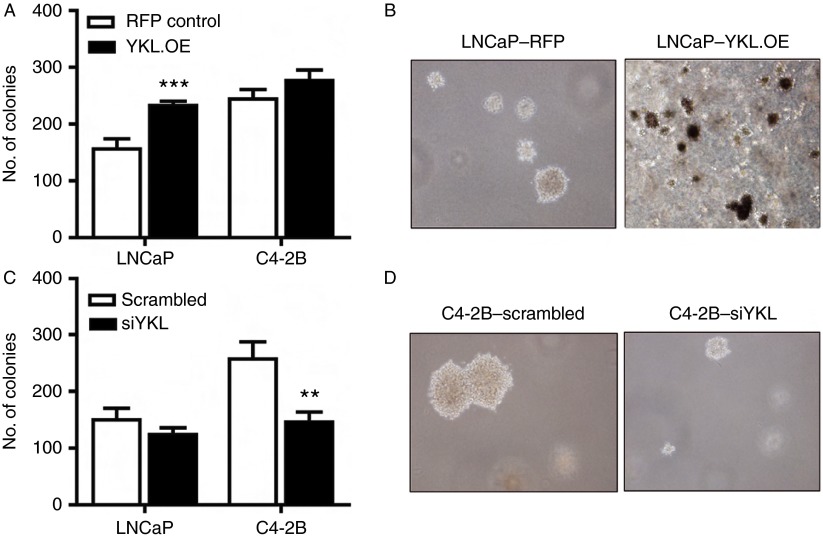
Effect of YKL40 on anchorage-independent growth: anchorage-independent growth of LNCaP and C4-2B cells was assessed by soft agar colony count assay. (A) YKL40-overexpressing (YKL.OE) and RFP control cells were plated in soft agar and the colony count was performed after 14 days. (B) Representative images of LNCaP–RFP vs YKL.OE cells, magnification 20×. (C) *YKL40*-silenced and scrambled control cells were seeded in soft agar and the colonies were counted after 2 weeks. (D) Representative photomicrographs of C4-2B cells±*YKL40* knockdown, magnification 20×. Data are expressed as mean±s.e.m., *n*=3. **P<0.01 and ***P<0.001. A full colour version of this figure is available at http://dx.doi.org/10.1530/ERC-14-0267.

As the ability of cells to grow in the absence of adhesion is a feature of malignant transformation, we investigated the effect of YKL40 on colony formation in soft agar. In LNCaP cells, YKL40 overexpression (YKL.OE) produced a significantly higher number of colonies when compared with the RFP control cells (*P*<0.001; Fig. 9A and B), whereas siYKL–LNCaP cells did not show any significant reduction (Fig. 9C). However, *YKL40* silenced C4-2B cells showed a significant decrease in the number of colonies when compared with the scrambled control (*P*<0.01; Fig. 9C and D), while YKL.OE C4-2B cells did show a slight, but insignificant, increase in the colony numbers (Fig. 9A). This result indicates that as C4-2B cells have a higher basal YKL40 expression, knockdown of *YKL40* resulted in the marked decrease in colony numbers, while overexpression of YKL40 in LNCaP cells enhanced the colony formation.

## Discussion

To our knowledge, this study provides persuasive evidence for a functional role for YKL40 in PCa for the first time. Although the credentials of YKL40 as a prognostic biomarker have been investigated in PCa, the role it plays in PCa pathobiology has not been previously explored. Our results show that YKL40 expression increased with progression to a CRPC phenotype in AR^+ve^, androgen-responsive cell lines. In addition, the tissue expression of YKL40 correlated with the aggressiveness and progression of clinical PCa. We also show that YKL40 affects cell invasion, migration and anchorage-independent growth of PCa cells *in vitro*. These findings have provided *in vitro* biological evidence as to how the elevated serum levels of YKL40 reported in PCa patients may contribute to the tumour progression and metastasis of PCa.

YKL40 has been reported as a promising biomarker in several cancer types and also in a number of inflammatory disorders (Johansen *et al*. 2006). It has been reported that 16–74% of patients had elevated levels of serum YKL40 at the time of first cancer diagnosis; however, the percentage increased from 39–83% at diagnosis in patients with metastatic cancer (Johansen *et al*. 2006). Despite the prognostic elucidation of YKL40 in a broad spectrum of cancers, the exact function of YKL40 is poorly understood. Accumulating evidence suggests that YKL40 is more than a prognostic biomarker and has the capacity to influence a wide range of cellular processes, most notably angiogenesis and metastasis. In fact, emerging interest in the focused targeting of YKL40 led to the development of a YKL40-neutralising antibody, which abrogated tumour angiogenesis and progression of the glioblastoma cells *in vitro* and *in vivo* using animal models (Faibish *et al*. 2011).

As the expression of YKL40 by PCa cells is unclear, we first analysed the expression pattern of YKL40 in PCa cell lines and tissues. We discovered that the expression of YKL40 was significantly increased in AR^+ve^ PCa cell lines as they progressed towards a castrate-resistant and metastatic phenotype when compared with AR^−ve^ PCa and normal prostate epithelial cells, suggesting that YKL40 may be regulated in an androgen-dependent manner in the PCa cell setting. Indeed, we confirmed that YKL40 was upregulated in both LNCaP and LNCaP-derived C4-2B cell line in response to androgens (DHT and R1881), whereas this androgen-induced YKL40 expression was inhibited via co-treatment of androgen-sensitive LNCaP cells with the anti-androgen, enzalutamide, an effect not observed in the castration-resistant C4-2B cell line. We then monitored the direct effect of *AR* silencing on YKL40 expression. Although the mRNA expression of *YKL40* decreased significantly after *AR* knockdown, we did not observe the similar effect at the protein level. This result suggests that the expression of YKL40 is likely to be regulated by other pathways outside of AR at the translational level. Indeed, YKL40 is known to be regulated by PI3K/AKT (Recklies *et al*. 2002) and JNK/ERK (Ling & Recklies 2004) and these pathways play a crucial role in prostate carcinogenesis (Rodriguez-Berriguete *et al*. 2012, Bitting & Armstrong 2013). The bidirectional crosstalk between the AR and AKT pathways has been shown to fuel the growth of castration-resistant PCa cells (Wang *et al*. 2007, Kaarbo *et al*. 2010, Chandarlapaty *et al*. 2011, Mulholland *et al*. 2011) and phospho-ERK has been identified as a potential link between the PI3K/AKT pathway and the AR axis (Thomas *et al*. 2013). Thus, it is possible that YKL40 is similarly regulated in PCa cells; however, this concept requires further investigation.

Our current study also elucidated the expression profile of YKL40 in PCa tissue specimens. We have demonstrated that levels of *YKL40* mRNA increased in higher TNM stages and Gleason grades of PCa when compared with the matched normal prostate tissue. This result is consistent with the previous clinical findings that serum levels of YKL40 correlate with the progression of PCa (Johansen *et al*. 2007, Kucur *et al*. 2008, Ozdemir *et al*. 2012). However, we did not find any significant differences in YKL40 levels among the Gleason grades, which supports the findings of Ozdemir *et al*. (2012) in PCa serum samples. One reason to explain the lack of association with Gleason grade may be the relatively small number of samples screened in our study, in addition to the fact that YKL40 may be selectively upregulated in the metastatic group of patients in such cases. We have also analysed *YKL40* gene expression in the publicly available microarray and RNA-sequencing datasets, which corroborate our findings that *YKL40* gene expression increases progressively with advanced stages of PCa. Thus, our study along with other studies has shown that high expression of YKL40 in PCa tissues is associated with a more aggressive phenotype with a high metastatic potential.

Cell migration and invasion together is a fundamental process underlying cellular processes, such as angiogenesis, embryonic development, immune response, metastasis and invasion of cancer cells. Based on the initial characterisation of YKL40 in cancer cell lines and PCa tissues, we investigated the role of YKL40 in processes underlying metastasis. We revealed that knockdown of *YKL40* in the bone metastatic C4-2B cells decreased both migration and invasion, whereas overexpression in less aggressive LNCaP cells rendered them more migratory and invasive. Our data are in accordance with multiple studies implicating the role of YKL40 in promoting tumour cell mobility and invasiveness accompanied with an increased metastatic potential (Johansen *et al*. 2006). Although we did not explore the mechanisms involved in this action of YKL40, others have proposed that YKL40 acts through the transcription factors, NFIX3 and STAT3, and through regulation of MMP2 to promote glioma cell invasion and migration (Ku *et al*. 2011, Singh *et al*. 2011). Interestingly, Stat3 has been shown to promote the metastatic progression of human PCa cells *in vitro* and *in vivo* (Abdulghani *et al*. 2008, Gu *et al*. 2010) and inhibition of Stat3 suppressed PCa cell growth and invasion (Sun *et al*. 2012). MMP2 also induces PCa cell migration upon activation by androgens via PI3K-dependent androgen receptor transactivation (Liao *et al*. 2003). Moreover, a recent study has shown that YKL40 stimulates the migration of colon cancer cells through the secretion of chemokines, IL8 and MCP1, through the MAPK signalling pathway (Kawada *et al*. 2012). In addition, YKL40 displayed the ability to enhance tumour metastasis via regulating the activation of pro-inflammatory cytokines in the animal models of metastatic breast cancer (Libreros *et al*. 2012). Together, these studies provide valuable information about the possible mechanisms of YKL40-mediated migration and invasion.

The ability of cancer cells to grow in the absence of adhesion to basement membrane, termed anchorage independence, correlates closely with tumourigenicity in animal models (Freedman & Shin 1974, Jiang & Liao 2004). Anchorage independence allows tumour cells to proliferate and invade adjacent tissues and to disseminate through the body, thus giving rise to metastasis. In a xenograft mouse model using human glioblastoma cell lines, YKL40 was shown to positively regulate tumourigenesis and metastasis (Francescone *et al*. 2011). Clonogenic growth assays also elucidated the ability of YKL40 to encourage anchorage-independent growth during invasion by inhibiting anoikis-related apoptotic pathways of glioma cells (Ku *et al*. 2011). Similarly, our results indicate that YKL40 suppression inhibits, and YKL40 overexpression increases, the anchorage-independent growth of PCa cells. Although molecular mechanisms responsible for this behaviour of YKL40 in PCa cells remain yet to be investigated, our preliminary results highlight the potential role of YKL40 in promoting tumourigenicity.

To conclude, our study characterises the role of YKL40 for the first time in PCa and provides valuable insights into its function in PCa cells. Collectively our results suggest that the development of a targeted agent against YKL40 may provide a novel strategy to inhibit the progression of metastatic PCa. Should such an agent reach clinical trials, measurement of serum levels of YKL40 may provide an opportunity to stratify patients, given the reliable detection of YKL40 in serum and thus optimising the efficacy of YKL40-targeted approaches.

## Supplementary data

This is linked to the online version of the paper at http://dx.doi.org/10.1530/ERC-14-0267.

## Author contribution statement

V Jeet conceived, designed and performed the experiments. G Tevz, M Lehman, B Hollier and C Nelson contributed reagents/materials/analysis tools. V Jeet wrote the paper. G Tevz, M Lehman, B Hollier and C Nelson edited the paper.

## Supplementary Material

Supplementary Data

## Figures and Tables

**Table 1:** Summary overview of patient cohorts used in this study

**Clinical characteristics**		**Relative YKL40 expression** (mean±s.d.)	***P* value** (normal vs tumour – stages and grades)
Patient's age (years)			
Age range at diagnosis or at tissue excision	46–87		
Mean age	62		
Percentage of cancer (%)	40–95		
Cells in tumour tissue			
Mean (%)	76		
Number of control samples			
Normal adjacent	17	1.25±0.8	
Number of tumour samples			
Stage T2	40	5.08±3.23	0.0483
Stage T3	30	7.89±2.23	0.0001
Stage T4	4	12.14±2.9	0.0035
Gleason 5 and 6	10	2.36±2.00	0.0631
Gleason 7	44	4.51±2.20	0.0073
Gleason 8	13	5.49±2.07	0.0028
Gleason 9	8	7.52±1.97	0.0035
