# Supplementary material for: Elevated YKL40 is associated with advanced prostate cancer (PCa) and positively regulates invasion and migration of PCa cells
Source: Endocr Relat Cancer. 2014 Jun 30;21(5):723–37. doi: 10.1530/ERC-14-0267 (PMC4134518; doi:10.1530/ERC-14-0267)
Supplement: Supplementary Data [file supp_21_5_723__index.html]

Elevated YKL40 is associated with advanced prostate cancer (PCa) and positively regulates invasion and migration of PCa cells — Role of YKL40 in prostate cancer — Supplementary Data 

# Elevated YKL40 is associated with advanced prostate cancer (PCa) and positively regulates invasion and migration of PCa cells

## Supplementary Data

**Files in this Data Supplement:**

- Supplementary Table 1 - (XLSX 45 KB)
- Supplementary Video 1 - (MP4 432 KB)
- Supplementary Video 2 - (MP4 494 KB)
- Supplementary Video 3 - (MP4 416 KB)
- Supplementary Video 4 - (MP4 427 KB)
- Supplementary Video 5 - (MP4 504 KB)
- Supplementary Video 6 - (MP4 579 KB)
- Supplementary Video 7 - (MP4 551 KB)
- Supplementary Video 8 - (MP4 537 KB)
- Supplementary Video 9 - (MP4 483 KB)
- Supplementary Video 10 - (MP4 533 KB)
- Supplementary Video 11 - (MP4 438 KB)
- Supplementary Video 12 - (MP4 385 KB)
- Supplementary Video 13 - (MP4 589 KB)
- Supplementary Video 14 - (MP4 599 KB)
- Supplementary Video 15 - (MP4 539 KB)
- Supplementary Video 16 - (MP4 571 KB)
